# Biphasic investigation of contact mechanics in natural human hips during activities

**DOI:** 10.1177/0954411914537617

**Published:** 2014-06

**Authors:** Junyan Li, Xijin Hua, Zhongmin Jin, John Fisher, Ruth K Wilcox

**Affiliations:** 1 Institute of Medical and Biological Engineering, School of Mechanical Engineering, University of Leeds, Leeds, UK; 2 School of Mechanical Engineering, Xi’an Jiaotong University, Xi’an, China

**Keywords:** Contact mechanics, articular cartilage, biphasic, fluid pressure, activities of daily living

## Abstract

The aim of this study was to determine the cartilage contact mechanics and the associated fluid pressurisation of the hip joint under eight daily activities, using a three-dimensional finite element hip model with biphasic cartilage layers and generic geometries. Loads with spatial and temporal variations were applied over time and the time-dependent performance of the hip cartilage during walking was also evaluated. It was found that the fluid support ratio was over 90% during the majority of the cycles for all the eight activities. A reduced fluid support ratio was observed for the time at which the contact region slid towards the interior edge of the acetabular cartilage, but these occurred when the absolute level of the peak contact stress was minimal. Over 10 cycles of gait, the peak contact stress and peak fluid pressure remained constant, but a faster process of fluid exudation was observed for the interior edge region of the acetabular cartilage. The results demonstrate the excellent function of the hip cartilage within which the solid matrix is prevented from high levels of stress during activities owing to the load shared by fluid pressurisation. The findings are important in gaining a better understanding of the hip function during daily activities, as well as the pathology of hip degeneration and potential for future interventions. They provide a basis for future subject-specific biphasic investigations of hip performance during activities.

## Introduction

Articular joints are remarkable bearings. Healthy articular joints are capable of withstanding many thousands of cycles of high load every year. For example, the human hip joint, which bears the weight of the upper extremity as well as the loads generated by the wrapping muscles, undergoes forces several times body weight even during normal activities such as walking. The excellent performance of such joints is a result of the articular cartilage, which comprises two principal phases: a solid phase composed of collagen fibrils and proteoglycan macromolecules and a water-like fluid phase. It has been well recognised that interstitial fluid pressurisation plays an essential role in load bearing and lubrication of articular cartilage^1–3^ and is closely linked with cartilage function and degradation (e.g. osteoarthritis).^4,5^ Consequently, these biphasic properties need to be taken into account to fully understand the function of the joint. The inclusion of such biphasic behaviour within a model would allow, for example, the interstitial fluid pressurisation under different activities to be determined, and the effect of changes in the cartilage properties caused by osteoarthritis or associated treatments to be evaluated.

The hip joint is subject to a range of loading directions and magnitudes during daily activities, which affect both its biphasic performance and the potential for degeneration. The contact pressure and area during different activities have been determined by a multitude of previous studies, through either experimental measurements^6–8^ or computational approaches.^9–11^ However, none of these studies have focused on the fluid pressurisation of the joint cartilage. Although several biphasic investigations for the hip joint have been conducted in two dimensions,^12–14^ or using hemiarthroplasty models,^15,16^ such methods have yet to be successfully applied to a three-dimensional (3D) whole joint under high physiological dynamic loads which involve consecutively sudden spatial and temporal variations, due to convergence difficulties.^17^ Using a new method, the authors have recently successfully simulated a whole hip model with biphasic cartilage layers under static physiological loads for a prolonged period.^18^ However, a biphasic study of the hip joint under dynamic physiological loads has yet to be reported. Such an investigation has the potential to provide valuable information about hip function and the potential for damage associated with daily activities. The aim of this study was to investigate the cartilage contact mechanics and the associated fluid pressurisation in the hip joint during different daily activities.

## Methods

**Figure 1.**
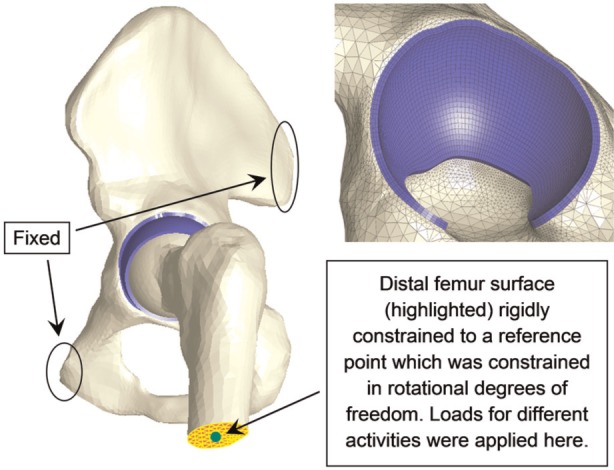
Finite element model of the hip joint with boundary conditions and the hexahedral mesh of the acetabular cartilage.

A previously published generic whole hip model was adopted (Figure 1).^18^ The modelling methodology was validated by comparing the contact area predicted from specimen-specific models of a porcine hip with experimental data on the same specimen; good agreement in location, shape and magnitude of contact area was achieved.^19,20^ Briefly, the articulating surfaces were assumed to be spherical^21,22^ with a uniform thickness of 2 mm for the cartilage.^23^ The radii for the articulating surfaces of the acetabulum and the femoral head were 28 and 27.5 mm, respectively.^8,24^ The bone and cartilage were represented by approximately 135,000 tetrahedral elements and 14,100 hexahedral elements, respectively. The cartilage and bone were bound together through sharing the same nodes on their interface. Mesh density was checked to ensure that the differences in the results of interest were less than 5% when the number of elements was doubled.

The cartilage was modelled as a biphasic solid and the solid phase was represented as neo-Hookean, with aggregate Young’s modulus E = 1.2 MPa, Poisson’s ratio ν = 0.045 and permeability K = 0.0009 mm^4^/N s.^25^ The material constitutive relationship was verified by comparing the predictions of indentation models using two different solvers, and nearly identical results were observed.^18^ The bone was modelled as impermeable and linearly elastic with Young’s modulus of 17,000 MPa and Poisson’s ratio of 0.3.^26^ The cortical bone and trabecular bone were modelled together because the difference in Young’s modulus between these two types of bone had little effect on the results.

**Figure 2.**
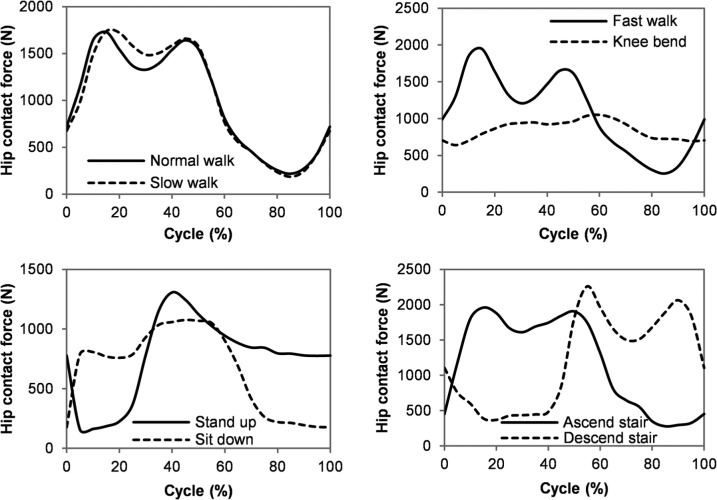
Hip contact force as model inputs, based on the average value of patient KW.^31^ Values of total force vector were presented.

The pelvis was fixed at the sacroiliac and pubis symphysis joints. The contact between articulating surfaces was assumed to be frictionless,^27^ as synovial joints have an extremely low coefficient of friction.^28^ The contact formulation allowed fluid to flow between contacting surfaces as well as from open surfaces of the cartilage.^29^ The distal surface of the femur was rigidly constrained to a reference point to which the boundary conditions for the femur were applied (Figure 1). It was assumed that the rotation of the femur would not cause a difference in the contact mechanics of the model because (1) the bone had little influence on the predictions, (2) the joint was assumed to be spherical and frictionless and (3) the long-term cartilage consolidation was not investigated in this study. Additionally, the minimal influence of rotation during short loading period was confirmed using simple 3D spherical arch-shaped hip models with only one element through the thickness (equivalent to two-dimensional (2D) models) in which the acetabular cartilage was fixed and the femoral head cartilage was loaded and rotated with a speed and range of rotation similar to the hip flexion/extension during gait. Minimal variations in the model predictions were observed over 10 cycles. Consequently, the femur was fixed along rotational degrees of freedom through the reference point. The femur was allowed to move freely along translational degrees of freedom to allow self-alignment. Previously measured in vivo hip contact forces for eight activities using instrumented total hip prosthesis^30,31^ were adopted and applied to the reference point on the distal femur (Figure 2 and Table 1). The directions and magnitudes of the loads were varied to represent time steps through the loading cycle. The lengths of time steps were checked to ensure that the results were insensitive to shorter steps.

**Table 1.** List of activities and their cycle durations (s).

Activity	Fast walk	Normal walk	Slow walk	Stand up	Sit down	Ascend stair	Descend stair	Knee bend
Time	1.0	1.1	1.2	2.5	3.7	1.7	1.5	3.6

Labrum exclusion was assumed in the present model due to a lack of extensive reports on its geometric and material parameters.^10^ The labrum is believed to provide little assistance in supporting load,^32^ but help impede fluid exudation through its lower permeability than the cartilage.^12–14,33^ To evaluate the sealing effect of the labrum, the edge surface of the cartilage was defined sequentially as free-draining and sealed. No obvious difference was detected for the short loading period.

All analyses were conducted using FEBio (version 1.5.0; Musculoskeletal Research Laboratories, Salt Lake City, UT, USA; URL: mrl.sci.utah.edu/software/febio)^29^ due to its good convergence capability in the simulation of biphasic materials in contact. Contact stress, fluid pressure and fluid support ratio for one cycle were recorded for each activity to evaluate the load transmission and tribological performance. The results of the walking activities were recorded for 10 cycles to investigate the time-dependent behaviour of the hip cartilage under dynamic loads. Due to the high computational expense involved in the simulation of biphasic materials, more gait cycles were not investigated. The number is comparable with previous models (e.g. six cycles in the hemiarthroplasty hip model^15^ and five cycles in the knee model^27^) and was sufficient to provide an initial insight into the time-dependent behaviour of the hip joint under dynamic loads.

## Results

**Figure 3.**
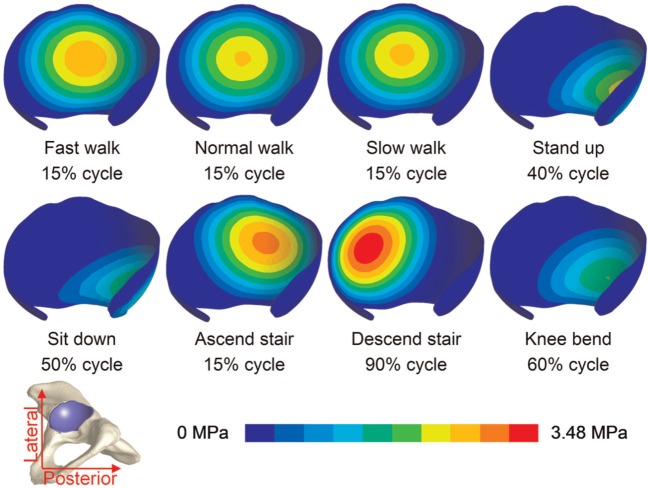
Contour of contact stress at % cycle for the time at which the peak value occurred. Among the different activities, the locations of contact varied, ranging from the central region to the posterior region of the acetabular cartilage.

In all loading cases, the magnitudes and distributions of fluid pressure and contact stress on the acetabular cartilage surface were very similar. The contact stress contours for all the eight activities for the time at which the peak value occurred are presented in Figure 3. At the occurrence of peak contact stress, the location of contact on the acetabular cartilage varied among the different activities, ranging from the central region for walking, ascending and descending stairs to the posterior region for standing up, sitting down and knee bending.

**Figure 4.**
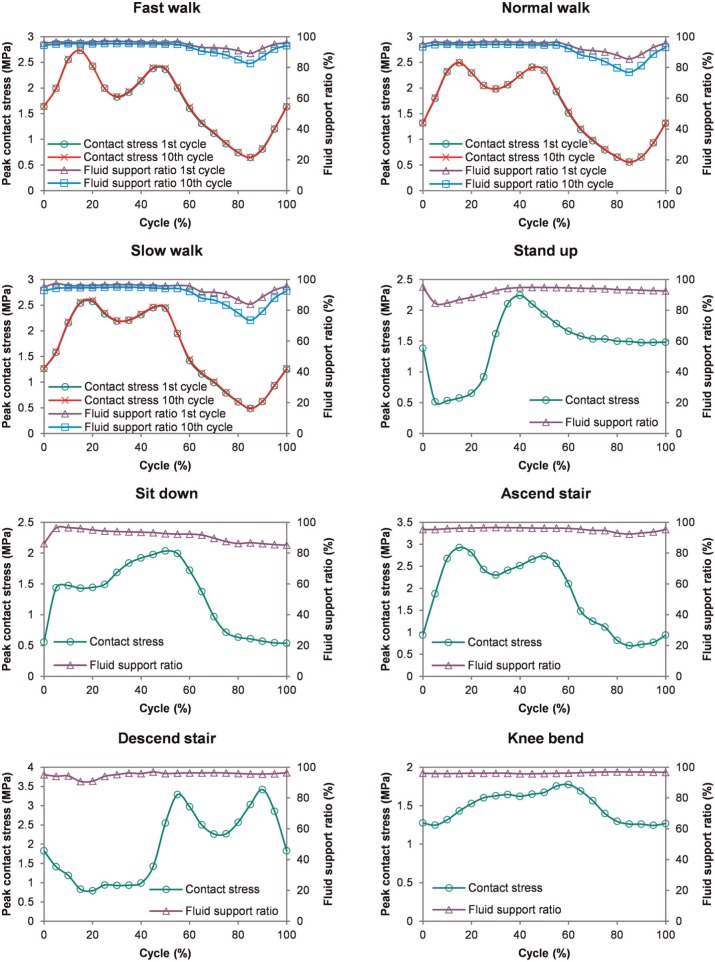
Peak contact stress and fluid support ratio of each cycle for all the activities. Results of 1st and 10th cycle of the walking activities were compared. For all the activities, the peak contact stress ranged from 1.8 to 3.5 MPa. The fluid support ratio was over 90% for the majority of a cycle of each activity but decreased below 90% at certain phases for some activities. The time-dependent behaviour of the joint cartilage over 10 cycles of gait was minimal.

The peak contact stress and fluid support ratio for each activity are presented in Figure 4. For all the activities, the peak contact stresses displayed similar trends to the load inputs (Figure 2), with the highest value of 3.5 MPa occurring during stair decent and the lowest value of 1.8 MPa during knee bending. The fluid support ratio was maintained at above 90% for the majority of the cycle for all the activities, but dropped below 90% at certain phases of the walking, standing up and sitting down cycles. During these periods of lower fluid load support ratio, the peak contact stresses were no more than 1 MPa.

**Figure 5.**
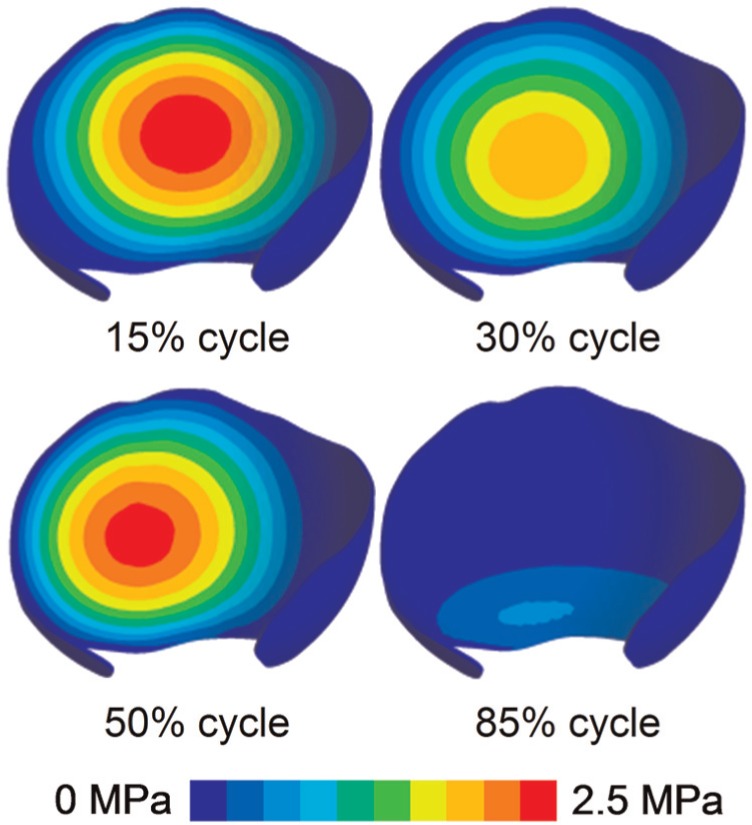
Contour of contact stress on the acetabular cartilage at different cycle phases of normal walk. Contact occurred around the central region during the majority of a cycle and slid towards the interior edge area during around 85% cycle.

**Figure 6.**
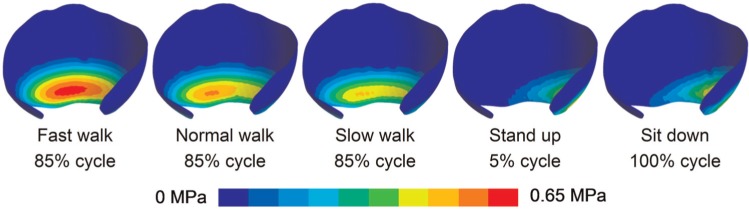
Contour of contact stress for activities when fluid support decreased below 90%. In all of such circumstances, the contact region was around the interior edge of the acetabular cartilage, and the stress level was low.

As an example to illustrate the relationship between the fluid support ratio and location of contact, the contact stress distribution at different phases of a cycle for normal walking is shown in Figure 5. The fluid support ratio was above 90% for the majority of a gait cycle when the contact region remained around the central area of the acetabular cartilage. However, it decreased markedly at around 85% of the cycle, when the minimum peak contact stress occurred and the contact region slid towards the interior edge of the acetabular cartilage. Similar patterns were found for other activities where the fluid support ratio decreased below 90% in that the contact occurred near the interior edge of the acetabular cartilage (Figure 6).

Over 10 cycles of walking, the peak fluid pressure and peak contact stress remained almost constant (<5%), while a reduction in fluid support ratio was detectable (Figure 4). This reduction was very minimal during the first 60% of the gait cycle, but became obvious during the latter 40% of the cycle when contact slid towards the interior edge of the acetabular cartilage.

## Discussion

In this study, the application of the biphasic cartilage properties enabled the investigation of the role that the interstitial fluid plays in the contact mechanic of the hip joint during daily activities. The predictions demonstrated that through pressurisation, fluid generally supported over 90% of the load transferred between the articulating surfaces of the hip joint for the daily activities investigated. Such high fluid support ratio would leave only a small portion load to the solid phase, causing a low level of solid phase stress and low friction coefficient that are essential for natural joint function.^4,5^ However, at certain stages for several activities, the fluid support ratio decreased below 90% when the contact region slid towards the interior edge of the acetabular cartilage, which is less confined than around the central region. This corresponds to previous studies which found that the fluid support ratio of the cartilage under unconfined compression is substantially lower than under confined compression.^34,35^ It can be concluded that the stress level of the solid matrix in the hip cartilage is linked with not only the magnitude but also location the contact stress. For the activities investigated in this study, the decrease in fluid support ratio led to an increased proportion of stress for the solid phase around the interior edge region of the acetabular cartilage, but the absolute magnitudes of the contact stress were very low (<1 MPa) at these stages, therefore potentially not harmful.

Over these 10 cycles, both the peak fluid pressure and contact stress remained nearly constant, suggesting that there was almost no variation in the fluid support ratio around the central region where peak fluid pressure/contact stress occurred. The fluid support ratio across the whole articulating surface decreased slightly, particularly when contact occurred around the interior edge of the acetabular cartilage. This is because the edge surface of the acetabular cartilage was free-draining, facilitating a substantially faster process of fluid exudation for the region nearby as compared with the central region of the acetabular cartilage. The faster fluid exudation process of the edge region may also contribute to the variation in the fluid support ratio across the whole articulating surface. Further studies involving more comprehensive and larger number of cycles of activities and potentially abnormal activities and anatomies are needed to better characterise the potential vulnerability of the interior edge region of the acetabular cartilage caused by the decreased fluid support ratio and faster fluid exudation process observed in this study.

Biphasic investigations have several advantages over single-phase assumptions, including the prediction of fluid pressurisation and time-dependent behaviour of the joint cartilage. Although as found by this study, the time-dependent response of the hip cartilage is not evident under instantaneous loads, it is not clear as yet whether single-phase representations are able to replicate the contact stress as predicted by biphasic simulations during short loading periods. Further studies will focus on systematic comparisons between single-phase and biphasic assumptions under varying circumstances (e.g. prolonged loading periods and dynamic loads of varying frequencies).

Although the modelling technique presented here represents an important step forward for whole joint investigations, there are several limitations. In this study, the solid matrix was assumed to be a homogeneous isotropic elastic material which may not fully represent the inhomogeneous fibre-reinforced structure of the cartilage.^2,36,37^ In reality, the fibre-reinforced structure may cause a substantially higher tensile stiffness than the aggregate stiffness of the cartilage.^38–40^ Consequently, the confinement effect due to the tensile stiffness may be reduced in the isotropic assumption, and the peak fluid pressure, peak contact stress and fluid support ratio may be underestimated. In particular, the level of reduction in fluid support ratio for the edge region of the acetabular cartilage may be overestimated. Further studies will be undertaken to investigate the influence of a tension–compression nonlinear solid phase on model predictions and computing expense, to determine whether it is necessary to incorporate this more sophisticated material behaviour. Although more cycles were investigated in this study than previous models,^15^ the period of 10 cycles of walking was not sufficient to allow all the outputs to have reached a stable state from one cycle to the next. In particular, the fluid support ratio during the latter part of the cycle was still dropping by the 10th cycle. The simulation was limited to 10 cycles by the restrictions in currently available resources, but increases in computational power will enable these changes to be investigated over more cycles in the future.

Another potential limitation is that an idealised generic geometry was assumed for the hip model with spherical articulating surfaces and uniform cartilage thickness, which has been shown to reduce the magnitude and distribution of the contact stress in an elastic finite element model.^41^ In this study, the peak contact stress was 2.5–3.5 MPa for a load of approximate 2000 N, which is lower than previous experimental studies.^6,8,10,42^ The higher values of such measurements are most likely to be caused by the idealised morphology and isotropic material assumptions used in this study. Also, the experimental measurement artefacts that arise from the highly conforming surfaces should be noted. For the purpose of this study, such assumptions were appropriate in order to compare hip performance among different activities and gain insight into the role of fluid pressurisation on the function of a generic hip joint. Finally, the labrum was excluded in this study. The sensitivity study reported in section ‘Methods’ demonstrated that there were no obvious differences between the models with a sealed and an open-edged surface of the cartilage over a short loading period. However, the labrum could also alter how loads are distributed over the surface of the cartilage. Therefore, the influence of the labrum on the biphasic behaviour of the hip should be further investigated and its geometry and properties characterised in more detail.

In conclusion, a generic human hip joint model was investigated in terms of its contact mechanics and associated fluid pressurisation during daily activities. Although several assumptions were made, the predictions provide insight into how the fluid pressurisation assists in hip function during daily activities. For all the activities investigated, the fluid supports most of the load transmitted between the articulating surfaces of the hip joint, thus playing an essential role in the lifetime survival of natural hip joints. A decreased level of fluid support ratio and a faster process of fluid exudation were observed for the interior edge region of the acetabular cartilage. The findings are important in better understanding the hip function during daily activities, as well as the pathology of hip degeneration. Further studies will focus on subject-specific modelling with more sophisticated material properties, enabling the models to be used to investigate how individual patient differences affect hip function.
